# Relationship Between Migraine and Alzheimer's Disease: A Study of Mendelian Randomization

**DOI:** 10.1002/brb3.71081

**Published:** 2026-01-25

**Authors:** Ran Bi, Weimin Zhao, Jiaoxuan Li, Songji Han, Haotian Qi, Jian Liu, Jinpu Wu, Shengxian Xu, Zhongda Sun, Zhenru Liu

**Affiliations:** ^1^ Changchun University of Traditional Chinese Medicine Changchun Jilin Province China; ^2^ Changchun University of Traditional Chinese Medicine Affiliated Hospital Changchun Jilin Province China

**Keywords:** Alzheimer's disease, migraine, Mendelian randomization

## Abstract

**Background:**

Given inconsistent evidence regarding the migraine‐Alzheimer's disease association, this Mendelian randomization (MR) study examines their potential causal relationship.

**Methods:**

GWAS summary statistics for migraine and Alzheimer's disease were acquired from the IEU Open GWAS repository. We implemented a multi‐stage MR framework comprising (1) univariate analysis, (2) independent replication, (3) multivariable MR, (4) meta‐analysis to evaluate migraine‐AD causality.

**Results:**

Univariate results showed OR = 13.43, 95% CI: 2.86‐63.16, *P <* 0.01; replication Mendelian randomization results were OR = 12.64, 2.89‐55.38, *P <* 0.01 with OR = 1.13, 95% CI: 1.06‐1.21, *P <* 0.01, meta‐analysis results were OR = 1.14, 95% CI: 1.07‐ 1.22, *P <* 0.01. Multivariate Mendelian randomization results were OR = 18.90, 95% CI: 1.69‐210.88, *P <* 0.01.

**Conclusion:**

Considering the observed epidemiological correlations and shared pathophysiological mechanisms between migraine and Alzheimer's disease (AD), we propose that chronic migraine may increase the susceptibility to AD through complex biological interactions. This hypothesis is reinforced by our Mendelian randomization (MR) findings, which support a causal relationship. Therefore, early and effective intervention in migraine management could serve as a promising strategy to mitigate the future risk of AD onset.

## Graphical abstract

Description of the design used in this Mendelian randomization (MR) study.

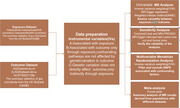

## 1 Introduction

Migraine is among the most widespread neurological conditions, impacting over one billion people worldwide. It typically presents with symptoms such as headaches, abdominal pain, nausea, and visual anomalies. As of 2016, migraine was identified as the second most common contributor to neurological disability‐adjusted life years (DALYs) on a global scale (Amiri et al. 2021). Its clinical course is frequently marked by disruptions in autonomic function, emotional regulation, cognition, and sensory perception—manifesting in features like muscle sensitivity and cutaneous allodynia (Lee et al. 2021).

Alzheimer's disease (AD), which represents the predominant form of dementia, currently affects more than 32 million individuals across the globe (Chi et al. 2014). Projections by the International Alzheimer's Association suggest that this figure may rise threefold by 2050 (Ding et al. 2020). Despite the looming public health challenge, the disease's onset and progression patterns remain poorly understood. Despite its growing public health impact, there remain substantial gaps in understanding the mechanisms underlying disease initiation and progression. Prior research has proposed several contributing pathological processes, including the deposition of β‐amyloid (Aβ), the development of neurofibrillary tangles containing hyperphosphorylated tau proteins, and dysregulated microglial activity in the central nervous system (Hane et al. 2017; Minter et al. 2016).

Despite extensive research on the migraine‐AD relationship, consensus remains elusive. Emerging evidence indicates migraine may increase AD risk (Chuang et al. 2013; Lee et al. 2019; Morton et al. 2019; Kostev et al. 2019; Cai et al. 2019). A study reporting elevated AD risk among migraineurs (RR = 1.33, 95% CI:1.16–1.53) (George et al. 2020). A large South Korean cohort finding 37% higher Alzheimer's dementia incidence (RR = 1.37, 95% CI:1.35–1.39) (Baars et al. 2010). However, contradictory results exist, including a multiethnic longitudinal study (*n* = 12,495; ages 51‐70) showing a non‐significant dementia association (RR = 1.04, 95% CI: 0.91–1.20) (Jelicic et al. 2000).

Despite the breadth of available research, most existing evidence stems from observational studies, which are inherently vulnerable to residual confounding and systematic biases. As a result, such designs face limitations in establishing definitive causal relationships. To overcome these challenges, we adopted a Mendelian randomization (MR) approach to explore the possible causal effect of migraine on Alzheimer's disease (AD). Mendelian randomization (MR) uses genetic variants, such as SNPs, as proxies for exposures to reduce confounding and reverse causation. The random allocation of these variants at conception mimics a natural experiment, similar to a randomized controlled trial in observational studies. (Jiang et al. 2022). Using public GWAS summary statistics, we conducted MR analyses to assess causal links between migraine and AD.

## 2 Methods

### 2.1 Study Design

MR is an instrumental variable analysis method based on genetic variation, mainly through single nucleotide polymorphisms (SNPs) as a proxy for modifiable exposure factors. To ensure the validity of the selected SNPs as instruments, three core assumptions must be satisfied (Jiang et al. 2022): (1) the selected SNPs demonstrate strong association with migraine (the exposure); (2) these genetic variants influence Alzheimer's disease risk exclusively through their effect on migraine (no pleiotropic effects); and (3) the instruments remain independent of any known or unknown confounders.

In this study, we initially assessed the causal relationship between the exposure (migraine) and the outcome (Alzheimer's disease) using univariate Mendelian randomization (MR) analysis. Subsequently, a meta‐analysis was conducted to confirm the consistency of the two‐sample MR results across multiple datasets. Finally, we applied multivariate MR analysis to adjust for potential confounding factors and enhance the robustness of causal inference.

### 2.2 Data Source

The pooled migraine GWAS data used in this study were derived from the IEU Open GWAS project (ebi‐a‐GCST90038646). The dataset is based on patient self‐reported diagnostic information and contains a total of 9,689,034 SNPs from 484,598 samples. (Kim et al., 2023).

Alzheimer's disease GWAS summary statistics from the latest large‐scale AD meta‐analysis (ebi‐a‐GCST90027158), comprising 111,326 individuals with clinically verified or proxy AD diagnoses and 677,663 controls (Emdin et al. 2017). These data were aggregated from European GWAS consortia and newly collected samples across 15 countries, resulting in a comprehensive pan‐European dataset.

All datasets were derived from publicly available sources and thus did not require ethical approval. All study participants were of European ancestry (see Table 1 for details).

**TABLE 1:** Information on exposure and outcome.

Characterization	Encodings	Sample size	Number of SNPs
Migraine	ebi‐a‐GCST90038646	484598	9587836
Migraine	ukb‐b‐16868	462,933	9,851,867
Migraine	finn‐b‐MIGRAINE_TRIPTAN	—	16,380,466
Alzheimer's disease	ebi‐a‐GCST90027158	487511	20921626

*Note*: Univariate MR exposure data code:ebi‐a‐GCST90038646.

Replication MR exposure data code:ukb‐b‐16868 and finn‐b‐MIGRAINE_TRIPTAN.

### 2.3 Univariate Mendelian Randomization Analysis

All SNPs included in this study met the criterion of *P* < 5 × 10^−^⁸. To mitigate potential bias arising from linkage disequilibrium (LD) between SNPs, we employed a reference threshold of LD *r* ^2^ < 0.001 and set the distance between each SNP to 10,000 kb to ensure their mutual independence.

To avoid weak instrument bias, instruments with F‐statistics < 10 were excluded. Following the above screening process, a total of 13 SNPs were ultimately included in this study.

Causal estimates were derived using inverse‐variance weighted (IVW), MR‐Egger, weighted median, and mode‐based (simple/weighted mode) methods.

One of the most important tests is the IVW method (Dönertaş et al. 2021)

### 2.4 Replication Mendelian Randomization and Meta‐Analysis

To address potential bias due to sample overlap, we employed a multicenter data integration approach. Specifically, migraine GWAS datasets were selected from two independent population cohorts: the UK Biobank (ukb‐b‐16868) and the Finnish Genetic Epidemiology Study (finn‐b‐MIGRAINE_TRIPTAN). By conducting independent analyses followed by meta‐analysis, we minimized the influence of population stratification and enhanced the generalizability of the findings across different populations. GWAS summary‐level data for migraine and proxy migraine used for replication were obtained from the IEU Open GWAS project. Specific dataset details are presented in Table 1.

### 2.5 Multivariate Mendelian Randomization Analysis

Multivariate MR (MVMR) permits the estimation of direct, independent effects of each exposure on the outcome by incorporating multiple instrumental variables (Bellenguez et al. 2022). Given the well‐documented associations between AD and metabolic/vascular factors (e.g., sex, BMI, hypertension, diabetes, dyslipidemia), our MVMR models incorporated covariates including body mass index, LDL cholesterol, triglycerides, hypertension status, and diabetes mellitus. (Leibson et al. 1997; Qiu et al. 2003; Reitz 2013; Lloret et al. 2019; Ding et al. 2020). All corresponding GWAS data were accessed through the IEU Open GWAS platform.

### 2.6 Sensitivity Analysis

To assess result reliability, we conducted several robustness checks: MR‐Egger for pleiotropy, MR‐PRESSO for outlier detection, Cochran's Q for heterogeneity, and leave‐one‐out analysis to examine SNP stability.

### 2.7 Statistical Analysis

Outcomes were expressed as ORs (binary) or β coefficients (continuous), both with 95% CIs, using *P <* 0.05 for statistical significance.

All analyses were conducted using the TwoSampleMR package in R.

## 3 Results

### 3.1 Univariate Mendelian Randomization

In this study, we went through a rigorous screening process and finally included 13 SNPs for final statistical analysis. The results of statistical analysis showed that IVW (OR = 13.43, 95% CI: 2.86‐63.16, *P <* 0.01), MR‐Egger (OR = 1.62, 95% CI: 0.004‐676.62, *P* = 0.88), and weighted median (OR = 34.83, 95% CI: 4.29‐63.16, *P <* 0.01) (See Table 2 for details).

**TABLE 2:** Mendelian randomization results.

Characterization	Encodings	OR	95% CI	p
IVW	ebi‐a‐GCST90038646	13.43	2.86‐63.16	<0.01
	ukb‐b‐16868	12.64	2.89‐55.38	<0.01
	finn‐b‐MIGRAINE_TRIPTAN	1.13	1.06‐1.21	<0.01
MR‐Egger	ebi‐a‐GCST90038646	1.62	0.004‐676.62	0.88
	ukb‐b‐16868	5.82	0.03‐1190.78	0.53
	finn‐b‐MIGRAINE_TRIPTAN	1.08	1.07‐1.28	0.71
Weighted median	ebi‐a‐GCST90038646	34.83	4.29‐63.16	<0.01
	ukb‐b‐16868	32.62	4.27‐249.19	<0.01
	finn‐b‐MIGRAINE_TRIPTAN	1.17	1.07‐1.28	<0.01

*Note*: Univariate MR exposure data code:ebi‐a‐GCST90038646.

Replication MR exposure data code:ukb‐b‐16868 and finn‐b‐MIGRAINE_TRIPTAN.

### 3.2 Heterogeneity and Pleiotropy Tests

**FIGURE 1:**
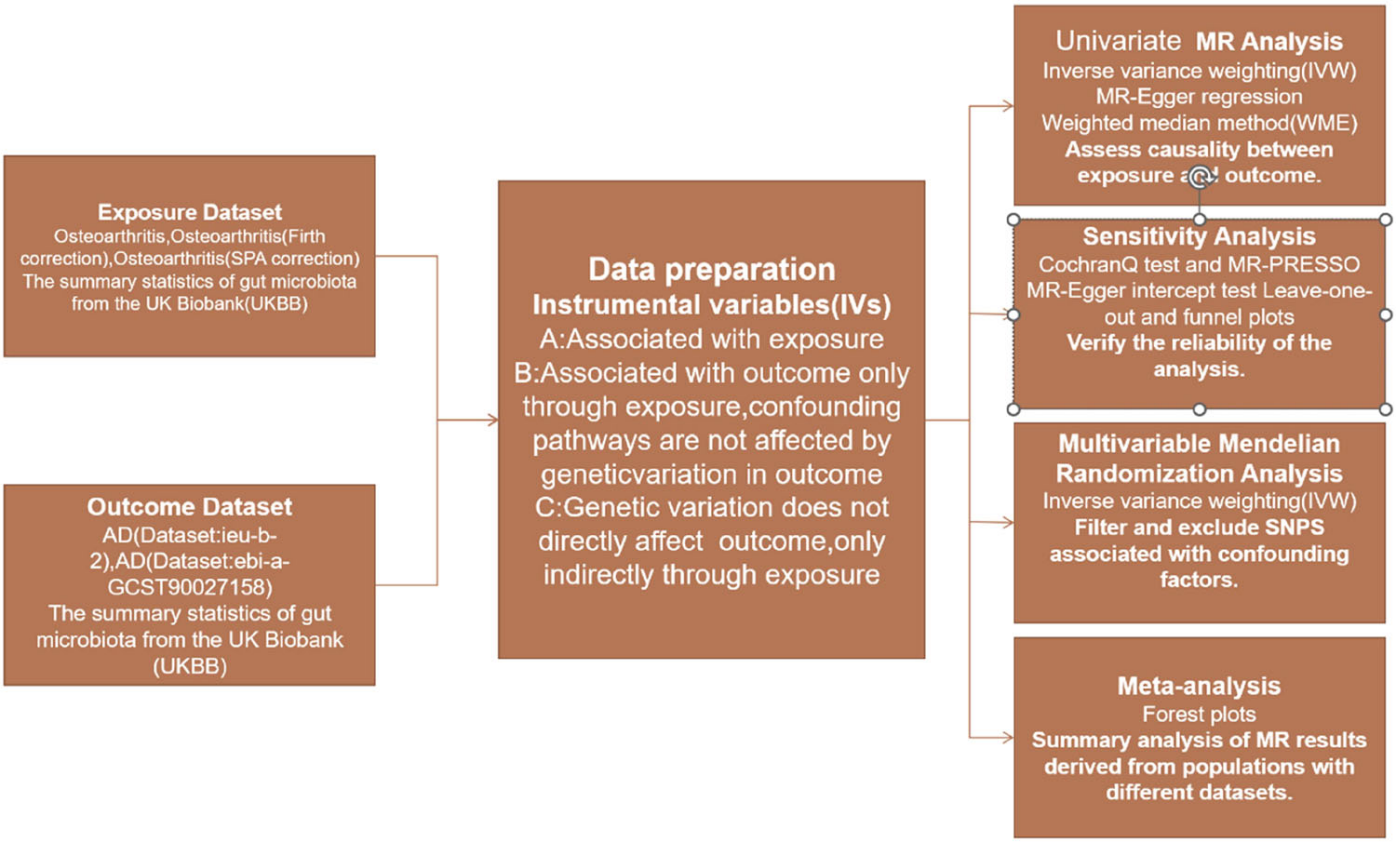
Mendelian randomization study design. Note: Univariate MR exposure data code:ebi‐a‐GCST90038646; Replication MR exposure data code: ukb‐b‐16868 and finn‐b‐MIGRAINE_TRIPTAN; SNP: single nucleotide polymorphism.

Sensitivity analyses showed that 1) MR‐Egger did not detect horizontal pleiotropy (intercept = 0.007, *P* > 0.05); 2) MR‐PRESSO did not detect abnormal outliers; and 3) Cochran's Q test suggested that there was no heterogeneity in instrumental variables (*P >* 0.05). Together, the above results confirmed the good stability of univariate Mendelian randomization analysis (see Table 3 for details). Figure 1

**TABLE 3:** Sensitivity analysis results.

	Heterogeneity test		MR‐Egger		MR‐PRESSO
Encodings	Cochran Q Statistics (df)	p	Intercept	p	p
ebi‐a‐GCST90038646	7.04 (11)	0.80	0.007	0.49	0.81
ukb‐b‐16868	9.50 (14)	0.80	0.002	0.77	0.79
finn‐b‐MIGRAINE_TRIPTAN	7.65 (8)	0.47	0.004	0.84	0.51

*Note*: Univariate MR exposure data code:ebi‐a‐GCST90038646.

Replication MR exposure data code:ukb‐b‐16868 and finn‐b‐MIGRAINE_TRIPTAN.

### 3.3 Replicating Mendelian Randomization

After stringent selection, 16 UK Biobank and 9 Finnish cohort SNPs were retained for MR analyses. Both cohorts showed consistent patterns:

- **IVW method** revealed significant associations (UKB: OR = 12.64, 95% CI = 2.89–55.38, *P <* 0.01; Finnish: OR = 1.13, 95% CI = 1.06–1.21, *P <* 0.01)
- **Weighted median** demonstrated strong effects (UKB: OR = 32.62, 95% CI = 4.27–249.19, *P <* 0.01; Finnish: OR = 1.17, 95% CI = 1.07–1.28, *P <* 0.01)
- **MR‐Egger** yielded non‐significant estimates (UKB: OR = 5.82, 95% CI = 0.03–1190.78, *P* = 0.53; Finnish: OR = 1.08, 95% CI = 0.73–1.61, *P* = 0.71)
  (See details for Table 2).

Sensitivity analyses uniformly indicated:

- No horizontal pleiotropy (MR‐Egger intercept: UKB = 0.003, *P* = 0.77; Finnish = 0.004, *P* = 0.84)
- Absence of outlier SNPs (MR‐PRESSO)
- No instrument heterogeneity (Cochran's Q: *P >* 0.05 for both)
  (See details for Table 3).

### 3.4 Meta‐Analysis

**FIGURE 2:**
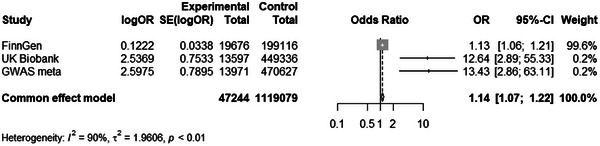
Meta‐analysis confirmed robust causality of migraine and Alzheimer's disease. Note: Univariate MR exposure data code: GWAS meta; Replication MR exposure data code: UK Biobank and FinnGen; The results of the meta‐analysis are as follows: Common effect model.

To make our results more robust, we subjected the results of the three Mendelian randomizations to meta‐analysis. In our study, we assessed heterogeneity between datasets, with *I* ^2^ = 90, indicating heterogeneity between our datasets. Therefore, we used a random‐effects model for subsequent analysis. Meta‐analysis confirmed robust IVW associations (OR = 1.14, 95% CI: 1.07–1.22, *P <* 0.01; Figure 2).

### 3.5 Multivariate Mendelian Randomization

Analysis based on multivariate Mendelian randomization (MVMR) showed a significant independent causal association between migraine and Alzheimer's disease after adjusting for relevant confounders (OR = 18.90, 95% CI: 1.69‐210.88, *P* = 0.02). This is consistent with our previous findings (see Table 4 for details).

**TABLE 4:** Results of multivariate Mendelian randomization.

Characterization	Encodings	OR	95% CI	P
Type 1 diabetes	ebi‐a‐GCST010681	1.02	0.08‐1.06	0.37
Type 2 diabetes	ebi‐a‐GCST006867	1.01	0.97‐1.06	0.59
High blood pressure	ebi‐a‐GCST90013916	0.91	0.86‐0.97	<0.01
Triglycerides LDL cholesterol HDL cholesterol Adult BMI females Migraine	ieu‐b‐111 ieu‐b‐5089 ebi‐a‐GCST90101746 ieu‐b‐5117 ebi‐a‐GCST90038646	1.01 0.87 1.06 0.82 18.90	0.93‐1.09 0.77‐0.99 1.00‐1.12 0.69‐0.98 1.69‐210.88	0.90 0.04 0.05 0.03 0.02

*Note*: The feature description column is the exposure factor included in multivariate Mendelian randomization.

## 4 Discussion

This research offers compelling genetic evidence indicating a causal association between migraine and Alzheimer's disease (AD), suggesting that migraine might serve as an independent risk contributor to the development of AD. These findings imply that persistent migraine could potentially elevate the long‐term likelihood of Alzheimer's onset.

Consistent with our results, prior Mendelian randomization (MR) investigations have implicated migraine as a likely contributor to Alzheimer's disease (AD) development (Geng and Chen 2025; Zhu et al. 2025). Neuroimaging assessments have identified reductions in hippocampal volume among individuals suffering from migraine, which may partly explain the increased susceptibility to Alzheimer‐related neurodegenerative changes (Cankaya et al. 2025). Additionally, large‐scale meta‐analyses based on population datasets have reinforced the hypothesis that migraine functions as an independent risk determinant for AD (Wang et al. 2022; Qu et al. 2022; Gu et al. 2022). Evidence from cohort studies further supports this link—for instance, Islamoska et al. observed that individuals diagnosed with migraine during middle age had a greater likelihood of developing AD later in life (Islamoska et al. 2020). Comparable findings were reported in Asian samples, where Chuang et al. demonstrated a significantly elevated dementia risk among migraine patients, even after adjusting for relevant confounders (Chuang et al. 2013).

Although the exact biological mechanisms that connect migraine to AD remain to be fully determined, existing evidence indicates that overlapping pathological processes—such as impaired insulin signaling within the brain and persistent neuroinflammation—may play a shared role. Brain insulin resistance, marked by reduced cellular responsiveness to insulin, has been proposed as a mechanistic link in both migraine pathophysiology (Kullmann et al. 2016; Mielke et al. 2005; Burgess et al. 2016) and AD progression (Nguyen et al. 2020; Yoon et al. 2023; Kellar and Craft 2020; Gupta et al. 2022). Disruptions in insulin pathways may contribute to abnormal protein aggregation, a hallmark of neurodegeneration.

Likewise, sustained neuroinflammatory activity is believed to be involved in the pathogenesis of both conditions. Elevated C‐reactive protein (CRP) levels have been frequently observed in migraineurs, suggesting systemic inflammatory involvement (Hagen et al. 2020; Hagen et al. 2019; Lippi et al. 2014; Geng et al. 2022). Inflammatory cytokines such as interleukin‐1β (IL‐1β), IL‐6, and tumor necrosis factor‐α (TNF‐α) are also commonly elevated during migraine attacks (Yücel et al. 2016; Wang et al. 2015; Perini et al. 2005; Armağan et al. 2020). These cytokines have been implicated in Alzheimer's pathology, particularly IL‐6, which has been closely associated with cognitive deterioration (Song et al. 2021; Long et al. 2023; Leonardo and Fregni 2023).

Considering the epidemiological links and shared pathophysiological characteristics between migraine and Alzheimer's disease (AD), we propose that chronic migraine could contribute to an increased risk of AD via multiple interrelated biological pathways. This hypothesis is supported by the outcomes of our Mendelian randomization (MR) analysis, which reinforce the possibility of a causal relationship. Therefore, early and effective management of migraine may represent a potential strategy for reducing the likelihood of developing Alzheimer's disease.

Although this study employed multiple methods to assess the robustness of the findings, several limitations remain. First, the analysis was based exclusively on European populations, making it unclear whether the results are generalizable to non‐European populations. Second, the study was based on limited summary‐level data and lacked access to individual‐level datasets, which may have influenced the precision of the results. Thirdly, there are significant differences in the odds ratios across various MR methods, particularly with the MR‐Egger analysis method. The MR‐Egger analysis method is primarily employed to estimate multiplicity effects and, under certain conditions, to estimate causality (its distinction from the IVW lies in accounting for weak instruments and intercepts, among other factors). Nevertheless, the IVW remains the primary approach. Additionally, wide confidence intervals were observed for some estimates, likely due to the use of weak instrumental variables and sample size limitations. Future studies should aim to improve the reliability of causal inference by increasing sample sizes, selecting stronger instruments, and potentially integrating multi‐omics data. Finally, this study did not conduct a bidirectional Mendelian randomization analysis. While existing epidemiological evidence suggests that migraine typically precedes the onset of Alzheimer's disease—and no reliable evidence supports the reverse—the possibility of reverse causation cannot be entirely excluded and warrants further investigation in future research.

## 5 Conclusions

The findings of this study offer genetic evidence suggesting a potential causal association between migraine and Alzheimer's disease (AD). Nonetheless, additional investigations are required to: (1) confirm these outcomes across varied ethnic and demographic groups; (2) further unravel the biological pathways that may mediate this link; and (3) better define the specific contribution of migraine to AD pathogenesis. Current research constraints underscore the necessity of large‐scale, multicenter studies to enhance the robustness, applicability, and mechanistic understanding of these associations.

## Author Contributions

**Weimin Zhao**: conceptualization, formal analysis, funding acquisition, writing – review and editing. **Ran Bi**: data curation, methodology, writing – original draft. **Jiaoxuan Li**: investigation. **Songji Han**: project administration. **Zhenru Liu**: resources. **Zhongda Sun**: software. **Shengxian Xu**: supervision. **Haotian Qi**: supervision, visualization. **Jinpu Wu**: validation. **Jian Liu**: visualization.

## Funding

The authors have nothing to report.

## Ethics Statement

Ethics committee approval is not required because this paper is a Mendelian randomization study using publicly available data and does not involve patients' personal data. Our research findings will be published in a professional academic journal.

## Conflicts of Interest

The authors declare no conflict of interest.

## Data Availability

All datasets were derived from publicly available sources and thus did not require ethical approval.
